# Collagen modifications predictive of lymph node metastasis in dogs with carcinoma in mixed tumours

**DOI:** 10.3389/fvets.2024.1362693

**Published:** 2024-03-06

**Authors:** Ana Paula Vargas Garcia, Daiana Yively Osorio Taborda, Luana Aparecida Reis, Ana Maria de Paula, Geovanni Dantas Cassali

**Affiliations:** ^1^Laboratory of Comparative Pathology, Institute of Biological Sciences, Federal University of Minas Gerais, Belo Horizonte, MG, Brazil; ^2^Biophotonics Laboratory, Physics Department, Institute of Exact Sciences, Federal University of Minas Gerais, Belo Horizonte, MG, Brazil

**Keywords:** canine mammary gland, tumour microenvironment, extracellular matrix, collagen fibres, breast cancer, mixed canine tumours, COX-2, metastatic disease

## Abstract

**Introduction:**

Mixed tumours in the canine mammary gland are the most common histological type in routine diagnosis. In general, these neoplasms have a favourable prognosis that does not evolve into metastatic disease. However, some cases develop into lymph node metastases and are associated with worse patient survival rates.

**Methods:**

Here is a retrospective study of 46 samples of primary mixed tumours of the canine mammary gland: 15 cases of benign mixed tumours (BMT), 16 cases of carcinoma in mixed tumours without lymph node metastasis (CMT), and 15 cases of carcinomas in mixed tumours with lymph node metastasis (CMTM). In addition, we selected 23 cases of normal mammary glands (NMT) for comparison. The samples were collected from biopsies performed during nodulectomy, simple mastectomy, regional mastectomy, or unilateral/bilateral radical mastectomy. We used multiphoton microscopy, second harmonic generation, and two-photon excited fluorescence, to evaluate the characteristics of collagen fibres and cellular components in biopsies stained with haematoxylin and eosin. We performed Ki67, ER, PR, and HER-2 immunostaining to define the immunophenotype and COX-2. We showed that carcinomas that evolved into metastatic disease (CMTM) present shorter and wavier collagen fibres as compared to CMT.

**Results and discussion:**

When compared to NMT and BMT the carcinomas present a smaller area of fibre coverage, a larger area of cellular coverage, and a larger number of individual fibres. Furthermore, we observed a correlation between the strong expression of COX-2 and a high rate of cell proliferation in carcinomas with a smaller area covered by cell fibres and a larger number of individual fibres. These findings highlight the fundamental role of collagen during tumour progression, especially in invasion and metastatic dissemination.

## 1 Introduction

Mixed tumours in canine mammary gland represent the most frequently diagnosed type in routine veterinary medicine diagnostics, accounting for 45.18% of mammary neoplasia cases (11.00% benign mixed tumours, 44.18% carcinomas in mixed tumours) according to a survey conducted in Brazil in 2018 (1). Carcinoma in mixed tumours (CMT) presents a complex histological pattern, as it contains epithelial and mesenchymal origin components. These tumours may exhibit pre-chondroid, chondroid, and osteoid matrix. Malignant epithelial cells show infiltrative growth identified by the loss of continuity of the basal/myoepithelial layer associated with clusters of tumour cells penetrating the stroma. Non-invasive proliferation can also be observed (1–3). The survival time of animals developing CMT can reach up to 1,800 days post-diagnosis, indicating a good prognosis. Although this histological type does not often develop into metastases, some cases progress to lymph node metastasis, which is associated with a shorter survival time (1–3).

The development of tumour cells is closely associated with changes in the microenvironment (4). Some of these changes result in a ‘hardening’ of the tumour-associated stroma, favouring tumour progression (4, 5). Alterations in the extracellular matrix (ECM) are linked to tumour progression and the metastatic process in mammary carcinomas (6–8). Previous studies have shown that intratumoural collagen properties are important for differentiating invasive tumours in mammary carcinomas in women (9–26), in dogs (27, 28), and specifically in canine mammary gland mixed tumours (29). A study published by our group (29) demonstrated that canine mammary carcinomas exhibit more organised collagen fibres as compared to healthy mammary gland tissue and that alterations in the length of collagen fibres directly correlate with tumour behaviour and animal survival time. Additionally, it was shown that benign, *in situ*, and invasive areas of CMT display significant differences in collagen fibres. Therefore, understanding the structure and functional properties of collagen fibres is essential for comprehending tumour behaviour in different types of neoplasms.

Histological grading analyses are useful for determining prognosis. However, due to the scarce areas of invasion in mixed tumours, it is extremely difficult to implement histological grading in most cases (1–3). Therefore, evaluating the tumour microenvironment concerning quantitative, organisational, and uniformity changes in collagen in these neoplasms can provide crucial information for the diagnosis, prognosis, and better support for affected patients. Therefore, the objective of this study was to identify changes in collagen fibres in canine mammary gland mixed tumour carcinomas with and without lymph node metastasis to correlate these findings with clinical and pathological characteristics.

## 2 Materials and methods

### 2.1 Selected samples

A retrospective study was conducted on 46 cases of canine mammary gland mixed tumours between the years 2011 and 2018, using biopsies on haematoxylin and eosin (H&E) stained slides. The cases were divided into 3 groups: 15 cases of benign mixed tumour (BMT), 16 cases of carcinoma in the mixed tumour without metastasis (CMT), and 15 cases of carcinoma in mixed tumour with metastasis (CMTM). The term “carcinoma in mixed tumour” used in this study, as per Cassali et al. (3), corresponds to the nomenclature “carcinoma - mixed type” proposed in Goldschmidt et al. (30). For the analysis of normal parameters, 23 slides of normal canine mammary tissue stained with H&E were included. The samples were obtained from the Laboratory of Comparative Pathology at the Institute of Biological Sciences, Federal University of Minas Gerais, MG-Brazil. The cases were collected from surgical removals of mammary gland neoplastic lesions performed at the Veterinary Hospital of the Federal University of Minas Gerais and sent to the laboratory for histopathological diagnosis. The samples were collected from biopsies performed during nodulectomy, simple mastectomy, regional mastectomy, or unilateral/bilateral radical mastectomy, depending on factors such as tumour size, clinical staging, lymphatic drainage, and tumour location. The neoplasms classification was carried out according to the criteria described in the Consensus for the Diagnosis, Prognosis, and Treatment of Canine Mammary Tumours (31) by a diplomate and experienced veterinary pathologist in canine mammary neoplasms. The clinical staging of carcinomas was performed according to the TNM system established by the World Health Organisation (WHO) and modified by Owen for the canine species (32). For carcinomas where there were at least 10 invasive areas, histological grading was determined according to the Nottingham system (31, 32).

### 2.2 Immunohistochemistry

The polymer detection system prepared histological sections of 4 μm thickness for immunohistochemical reactions. A commercial anti-mouse/anti-rabbit detection kit (Novolink Polymer Detection System, Leica Biosystems, Newcastle Upon Tyne, United Kingdom) was used following the manufacturer’s instructions. Antigen retrieval for oestrogen receptors (ER), progesterone receptors (PR), Ki67, Cyclooxygenase-2 (COX2), and human epidermal growth factor receptor 2 (HER2) was achieved using steam heat (Pascal^®^) with citrate at pH 6.0 (Dako Cytomation Target Retrieval Solution, Dako, Glostrup, Denmark). Slides with histological sections were incubated with the appropriate primary antibody for 16 h in a humid chamber at 4°C (1,50, clone 1D5, Dako for ER; 1:50, clone hPRa2, Neomarkers for PR; 1:200, polyclonal, Dako for HER2; 1:50, clone MIB-1, Dako for Ki67; and 1:50, clone SP21, Invitrogen for COX2). Immunoreactivity was visualised using the 3′-diaminobenzidine chromogen (DAB Substrate System, Dako, Carpinteria, CA, United States) and counterstained with Mayer’s haematoxylin. Fragments of mammary tissue positive for ER, PR, HER-2, COX2, and Ki67 were used as positive controls. For negative controls, the primary antibody was replaced with a phosphate-buffered saline (PBS) solution. The slides evaluation, the immunoreactive quantification, and the immunophenotypes classification were carried out by the criteria established by Nunes et al. (33). The antibodies used were standardised in our laboratory routine, and their antigenic specificity was confirmed through our research and by reference to other published studies (3, 28, 29, 33–38).

### 2.3 Multiphoton microscopy

Second Harmonic Generation microscopy (SHG) and Two-Photon Excited Fluorescence (TPEF) were performed at the Biophotonics Laboratory, Physics Department, Institute of Exact Sciences, Federal University of Minas Gerais. We used an Olympus FV300 laser scanning confocal system modified to excite the samples with a 800 nm wavelength Ti_sapphire laser beam. The experimental setup and image acquisition were performed following the protocols outlined by Reis et al. (28) and Garcia et al. (29). For measurements, 10 to 15 representative areas were selected from each sample. Normal mammary tissue (NMT) served as a normative parameter for comparison with benign and carcinomatous neoplastic cases. Due to the heterogeneity of the evaluated tumours, areas with abundant mesenchymal components were avoided.

### 2.4 Assessment of collagen fibres

For the quantitative analysis of collagen fibre parameters, the Python-based software *PyFibre - Python Fibrous Image Analysis Toolkit* (39) was employed, as previously performed by Reis et al. (28), and Garcia et al. (29). Metrics values assessed in histological sections from normal mammary tissue were used as a reference for comparison among other cases.

### 2.5 Statistical analysis

The assessment of normality in smaller sample groups was conducted using the Shapiro–Wilk test, whereas, for larger sample groups, the Kolmogorov–Smirnov test was employed. Considering the non-parametric distribution of the dataset, we utilised the Kruskal-Wallis test followed by the Dunn test to conduct multiple median comparisons among groups. We conducted Pearson correlation analyses for parametric data and Spearman for non-parametric data. Specific survival rate was estimated using Kaplan–Meier curve, and group comparisons were performed using the Mantel–Cox log-rank test. Analyses were conducted using Prism^®^ software (version 8.0, GraphPad^®^, San Diego, CA, United States) for the Microsoft^®^ Windows^®^ operating system. A value of *p* < 0.05 was considered statistically significant for all analyses.

### 2.6 Ethical considerations

The study was conducted following the fundamental ethical principles of Law No. 11,794 of October 8, 2008, and Decree No. 6,899 of July 2009, as well as the regulations issued by the National Council for Control of Animal Experimentation (CONCEA). It was approved by the ‘Ethics Committee for Animal Use’ at the Federal University of Minas Gerais under number 251/2018.

## 3 Results

### 3.1 Sample characterisation

Characteristics such as patient age, histological grade, immunophenotype, and survival time are presented in Table 1. A total of 46 cases were selected: 32.6% BMT (*n* = 15), 34.7% CMT (*n* = 16), and 32.6% CMTM (*n* = 15).

**Table 1:** Clinicopathological features of mixed tumour of the canine mammary gland.

Mixed tumour subtype	N	Middle Ages	Histological grading				Immunophenotype					Survival (days)
			I	II	III	NA	HR+/ HER2- Ki67 < 20%	HR+/ HER2-Ki67 > 20%	HR−/ HER2+	TN	NA	
BMT	15	11,4	0	0	0	15	13	0	0	0	2	NA
CMT	16	11,3	5	2	0	9	13	1	0	0	2	833
CMTM	15	10,4	7	2	0	6	10	2	2	0	1	691

Benign mixed tumour (BMT), carcinoma in mixed tumour without metastasis (CMT), carcinoma in mixed tumour with metastasis (CMTM). *N* = number of cases. NA was not evaluated. HR = hormonal receptor. Ki67 = cell proliferation. TN = triple negative (HR-/HER2-). It was not possible to grade 9 CMT and 6 CMTM because 10 invasive areas in the epithelial component were not identified for histological grading.

### 3.2 Image analysis and extracted parameters

**Figure 1:**
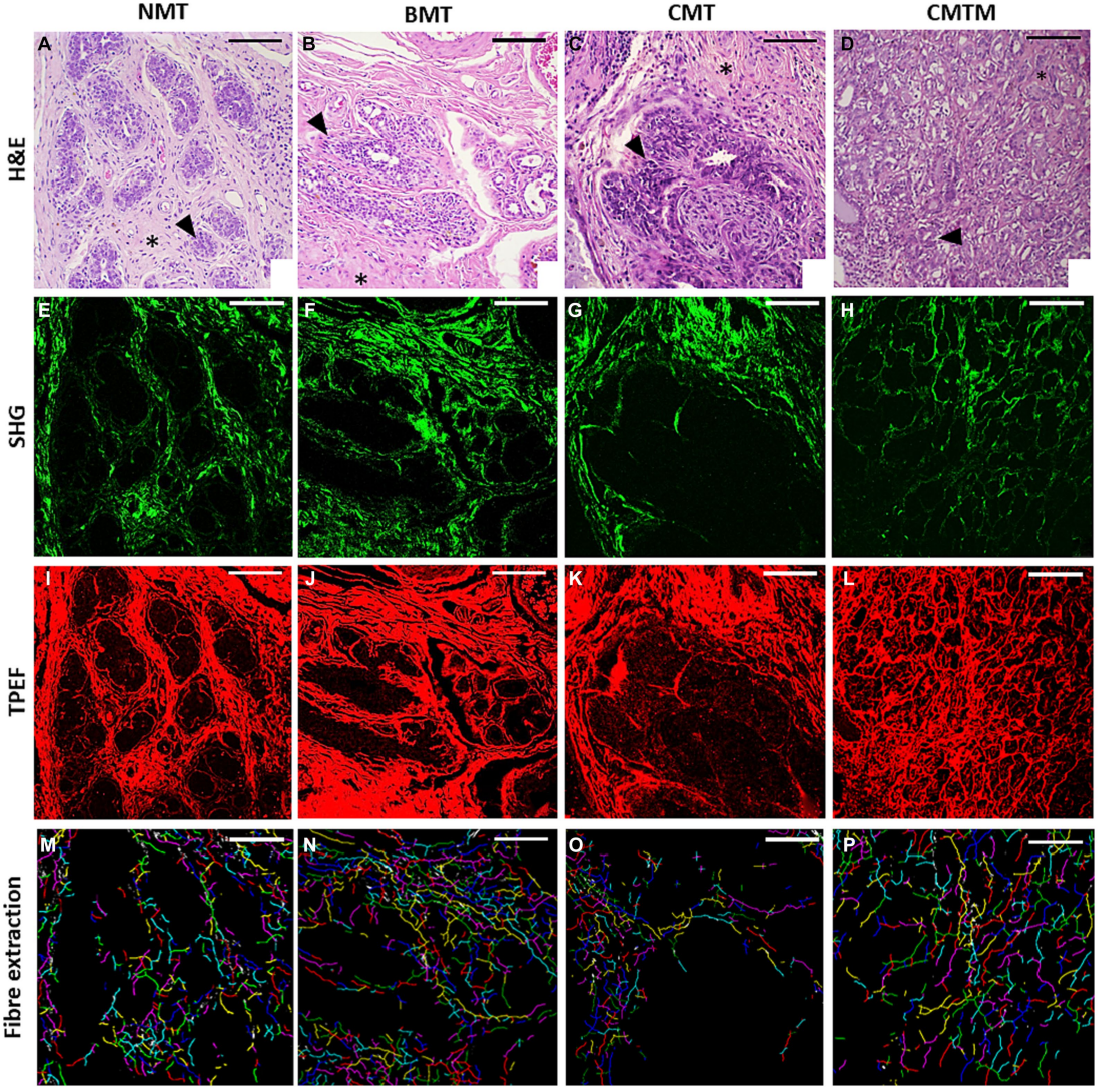
Acquired images (x20). The optical microscopy H&E **(A–D)**, second harmonic generation microscopy **(E–H)**, two-photon excited fluorescence microscopy **(I–L)**, and the extracted collagen fibres with each fibre in a random colour **(M–P)** for the canine normal mammary tissue **(A,E,I,M)**, benign mixed tumour **(B,F,J,N)**, carcinoma in the mixed tumour without metastasis **(C,G,K,O)** and carcinoma in mixed tumour with metastasis **(D,H,L,P)**. HE asterisks: stroma. HE arrowhead: epithelial cells. The scale bar is 100 μm.

Figure 1 shows representative images of histological sections from canine mammary gland samples examined in this study. In the columns, the histological types are denoted as NMT, BMT, CMT, and CMTM. Representative H&E stained slides were used for the evaluation of collagen fibres and cellular characteristics in each case (first row). The SHG signal (represented in the second column in a green colormap) is acquired from the same area, resulting exclusively from the structural conformation of the collagen fibres, making them the exclusive source of this signal in the tissue examined. Lastly, the TPEF images consist of signals emitted by the cellular component stained with eosin (third row, in a red colormap). Subsequently, the programme overlays the collected signals to automatically assess cellular components and collagen fibres, extracting the six selected metrics for this study: cell segment coverage, fibre segment coverage, fibre orientation (fibre segment SHG coherence), number of fibres (no. fibre), and mean fibre length. Our image analysis approach allowed the quantification of several visual features. In the fourth line, we observe the individual fibres represented in different random colours, emphasising the smaller number of fibres in the CMT and CMTM.

The normal mammary gland (first column) exhibits acini and ducts made up of epithelial cells lined by myoepithelial cells, separated from the adjacent connective tissue by the basement membrane. Throughout mammary tissue, collagen fibres are remarkably abundant in the surrounding connective tissue, exhibiting diverse orientations (40, 41). Benign mixed tumours (BMT), presented in the second column, manifest as the benign proliferation of cells that morphologically resemble normal epithelial cells and mesenchymal elements, generating cartilage, bone, adipose tissue, and possibly fibrous tissue. In these neoplasms, the surrounding connective tissue still contains a substantial amount of collagen fibres, although it presents a more organised arrangement as compared to NMT (1–3). The CMT and CMTM, presented in the third and fourth columns, respectively, exhibit a complex histological composition with elements derived from epithelial and mesenchymal origin. Notably, we did not observe significant differences in the histological pattern between carcinomas with and without metastasis (1–3).

### 3.3 Immunohistochemical evaluation of mixed tumours

**Figure 2:**
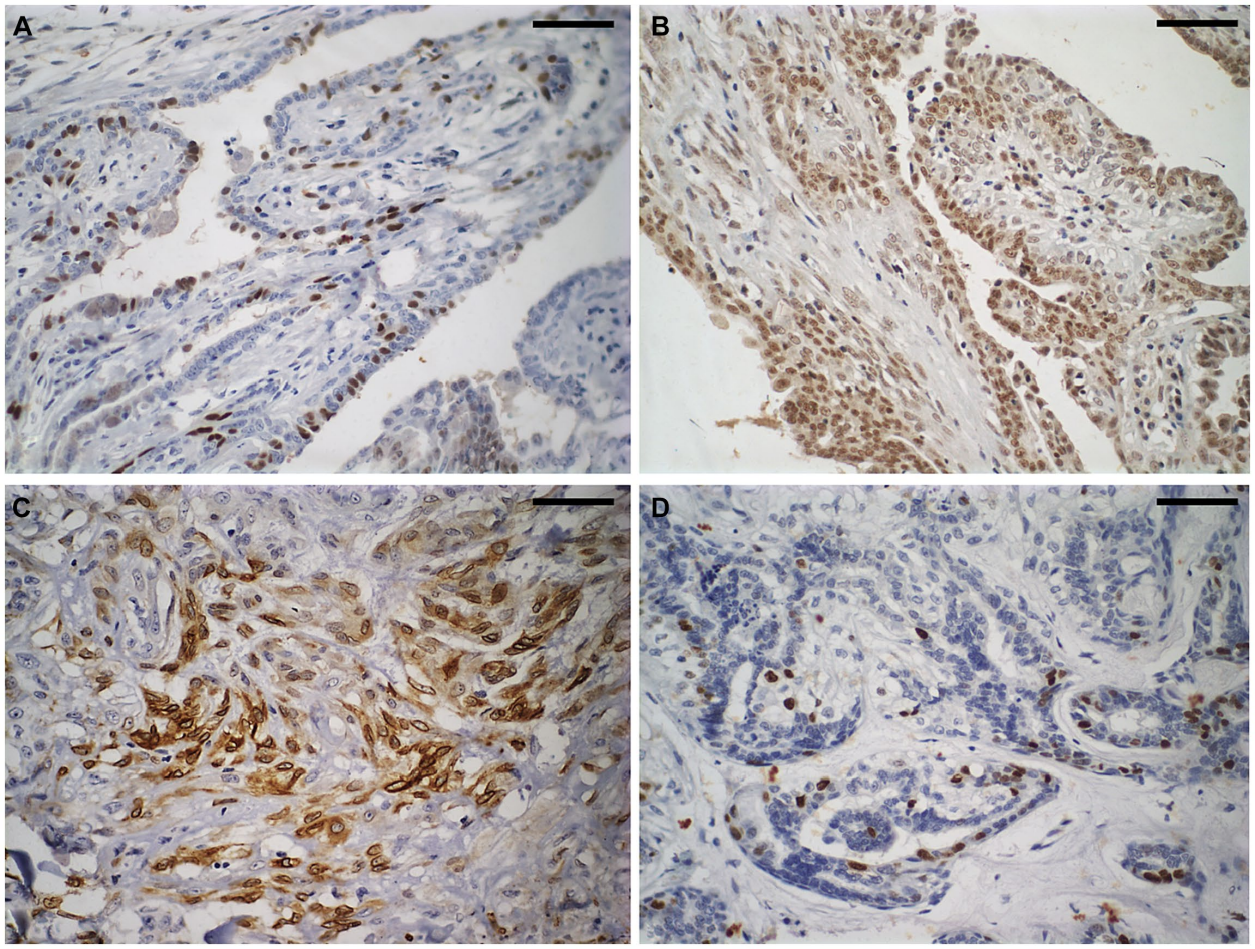
Immunostaining to evaluate the molecular subtype of carcinomas in mixed tumours of the canine mammary gland. Shown here is a carcinoma in a mixed tumour without lymph node metastasis. **(A)** Strong nuclear immunostaining for oestrogen receptor in approximately 25% of neoplastic cells. Diaminobenzidine chromogen, haematoxylin counterstaining. **(B)** Strong nuclear immunostaining for progesterone receptor (brown areas) in more than 75% of neoplastic cells. Diaminobenzidine chromogen, haematoxylin counterstaining. **(C)** Strong cytoplasmic immunostaining for cyclooxygenase-2 in approximately 60% of neoplastic cells. Diaminobenzidine chromogen, haematoxylin counterstaining. **(D)** Strong nuclear immunostaining for Ki67 in approximately 10% of neoplastic cells. Diaminobenzidine chromogen, haematoxylin counterstaining. The scale bar is 50 μm.

Figure 2 shows of immunostaining for COX2, Ki67, ER, and PR was conducted in BMT, CMT, and CMTM. Table 2 show the evaluation of immunohistochemical markers COX2, Ki67, HER2, ER, and PR was conducted in BMT, CMT, and CMTM. We established correlations between all metrics obtained from the excitation multiphoton microscopy form for the CMT and CMTM and the immunohistochemical data. These results are shown in Table 2.

**Table 2:** Immunophenotypic features of canine mammary neoplasms.

Mixed tumour subtype	COX2 (score =/> 6)			Ki67			HER2			Oestrogen receptor			Progesterone receptor		
	(+)	(−)	NA	>20%	<20%	NA	(+)	(−)	NA	(+)	(−)	NA	(+)	(−)	NA
BMT	0	11	4	0	13	2	0	13	2	13	0	2	13	0	2
CMT	4	10	2	1	12	2	0	14	1	13	0	2	13	0	2
CMTM	2	12	1	4	10	1	0	13	2	12	2	1	12	2	1

Benign mixed tumour (BMT), carcinoma in mixed tumour without metastasis (CMT), carcinoma in mixed tumour with metastasis (CMTM). HER2: human epidermal growth factor receptor 2. (+) = positive. (−) = negative. NA was not evaluated.

### 3.4 Carcinomas exhibit more organised collagen fibres

**Figure 3:**
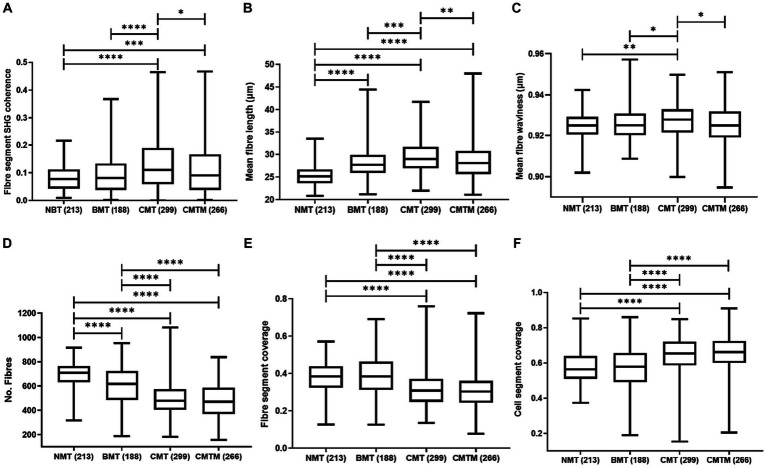
Parameters of the canine mammary gland were analysed by second harmonic generation and two-photon excited fluorescence techniques. Kruskal Wallis test and the Dunn test. Boxplots graphics showing the calculated parameters for the fibre segment SHG coherence **(A)**, mean fibre length **(B)**, mean fibre waviness **(C)**, no fibres **(D)**, fibre segment coverage **(E)**, and cell segment coverage **(F)** for canine. The centre lines show the medians, the box limits indicate the 25th and 75th percentiles and the whiskers extend 1.5 times the interquartile range from the 25th and 75th percentiles. The *p* values less than 0.001 are denoted by (***), *p* less than 0.01 are denoted by (**) and *p* less than (0.05) are denoted by (*).

Figure 3A shows the outcomes of the assessment of collagen fibre orientation (fibre segment SHG coherence) among the NMT, BMT, CMT, and CMTM. The collagen fibre organisation is a measure of the degree of alignment of collagen fibres, ranging from zero to one, where zero relates to randomly oriented fibres and one to fibres oriented in a single direction within the tissue. The orientation of collagen fibres in NMT showed no significant differences when compared to the fibre orientation in BMT. When contrasting BMT and CMT, the fibres appear more organised in CMT (*p* < 0.0001). For the comparison between NMT and CMT, the fibres present greater organisation in CMT (*p* < 0.0001). For NMT and CMTM, the fibres present greater organisation in CMT (*p* = 0.001). In tumours associated with lymph node metastasis (CMTM), we note that the fibre orientation is similar to that found in NMT and appears more disorganised compared to CMT (*p* = 0.0143).

### 3.5 Shorter and more wavy collagen fibres are associated with the presence of lymph node metastases

Figure 3B shows the mean fibre length of collagen fibres in NMT, BMT, CMT, and CMTM. This parameter measures the mean fibre length of collagen fibres in micrometres (μm). We observed that NMT exhibits shorter fibres as compared to BMT, CMT, and CMTM (*p* < 0.0001). There were no significant differences in fibre length between BMT and CMTM. BMT presents shorter fibres than CMT (*p* = 0.0005). When comparing the carcinomas, we note that CMT exhibits longer fibres as compared to CMTM (*p* = 0.0330).

Figure 3C shows the mean fibre waviness of collagen fibres in NMT, BMT, CMT, and CMTM. This parameter measures the waviness of collagen fibres, ranging from zero to one, where zero relates to more wavy fibres and one to more linearized fibres within the tissue. We observed that NMT presents more wavy fibres as compared to CMT (*p* = 0.003) and fibres with similar waviness to BMT and CMTM. BMT presents more wavy fibres as compared to CMT (*p* = 0.0410) and fibres with similar waviness to NMT and CMTM. CMT presents more linearized fibres compared to CMTM (*p* = 0.0294).

### 3.6 CTM and CTMM present higher collagen density and lower cell density

Figures 3D–F show the measurement of the number of fibres, fibre-covered area, and cell-covered area in NMT, BMT, CMT, and CMTM, respectively. Figure 3D shows that NMT exhibits a higher number of fibres when compared to BMT, CMT, and CMTM (*p* < 0.0001). BMT showed a greater number of fibres as compared to carcinomas (*p* < 0.0001). CMT and CMTM showed no significant differences in the number of fibres. Figures 3E,F present the areas covered by fibres and cells, respectively. We observed that carcinomas show similar proportions of the number of areas covered by collagen fibres. NMT and BMT showed no significant differences. NMT exhibits a larger quantity of areas covered by collagen fibres when compared to CMT and CMTM (*p* < 0.0001). Regarding the cell-covered area, NMT shows no significant differences when compared to BMT. NMT shows a smaller cell-covered area compared to the carcinomas (*p* < 0.0001). No significant differences were found in the cell segment coverage between CMT and CMTM.

### 3.7 Carcinomas with shorter collagen fibres have a greater number of fibres

**Figure 4:**
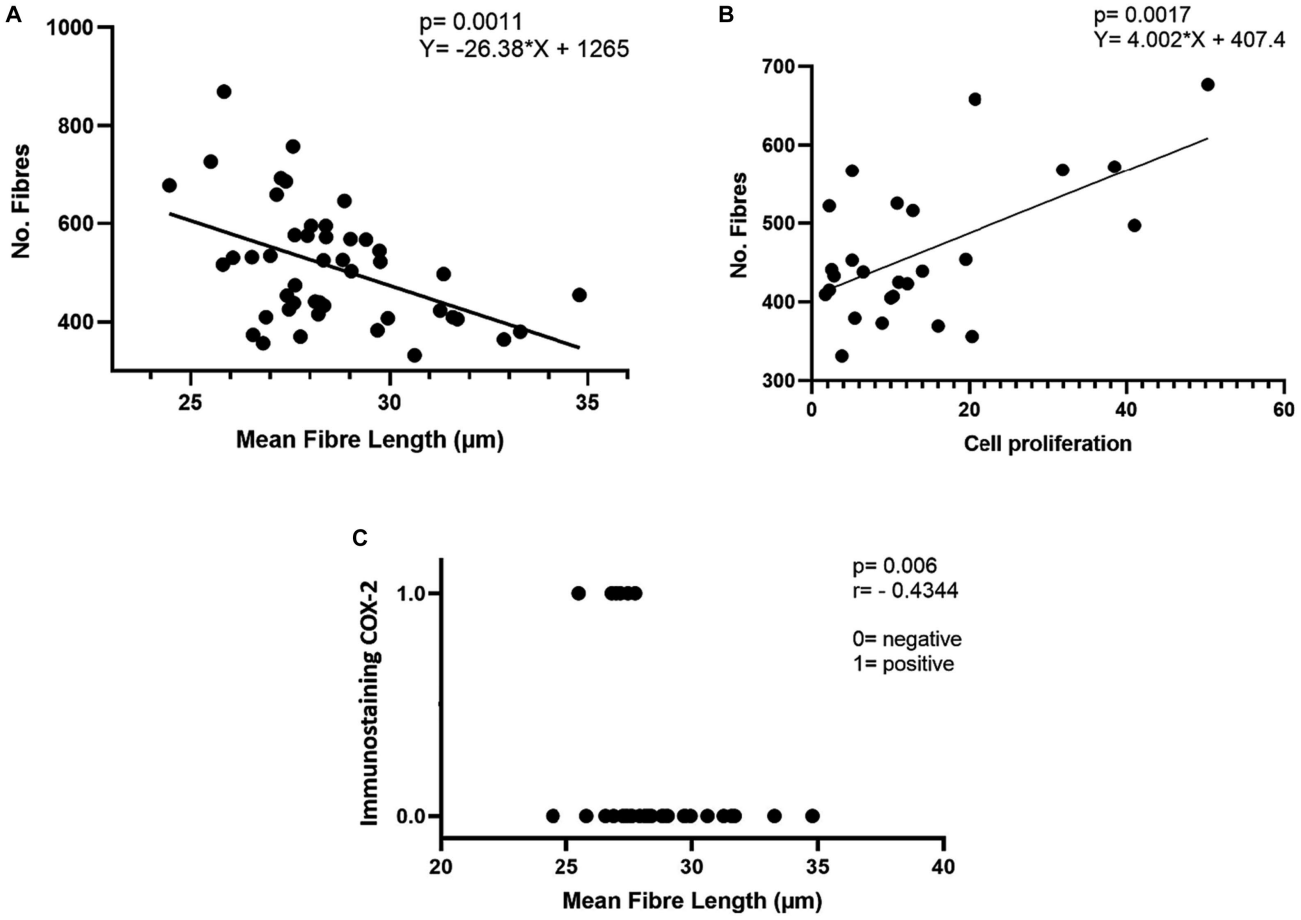
Correlation between immunostaining profile and collagen parameters evaluated in carcinomas in mixed tumours. **(A)** Linear regression between mean fibre length (μm) and a number of fibres in mixed tumours. **(B)** Linear regression between cell proliferation and number of fibres in mixed tumours. **(C)** Spearman’s correlation between mean fibre length (μm) and immunostaining for COX-2.

Figure 4A shows the linear regression between mean fibre length (μm) and the number of fibres in mixed tumours. The data show an inverse correlation between the mean length of collagen fibres and the number of collagen fibres in CMT and CMTM (*p* = 0.0011).

### 3.8 Immunohistochemical and collagen fibre correlations

#### 3.8.1 Carcinomas with higher proliferative rates present a larger number of fibres

Figure 4B shows the linear regression between cell proliferation and the number of fibres in mixed tumours. There is a direct correlation between cell proliferation and the number of collagen fibres in CMT and CMTM (*p* = 0.0001).

#### 3.8.2 Carcinomas with high expression of COX2 present smaller collagen fibres

Figure 4C shows the correlation between COX2 expression and the mean collagen fibre length. The data indicate that strong COX2 immunostaining in more than 30% of the tumours correlates inversely with the mean fibre length in mixed tumours (*p* = 0.006). For this assessment, two groups were considered: cases with a COX2 distribution equal to or less than 30% (0 on the y-axis) and cases with a COX2 distribution larger than 30% (1 on the y-axis).

All metrics obtained from the multiphoton microscopy for the CMT and CMTM were correlated with the immunohistochemical data, but no other statistically significant correlations were found.

## 4 Discussion

The tumour microenvironment (TME) comprises all elements that interact with cancer cells. TME consists of cellular components such as fibroblasts, endothelial cells, neurons, adipocytes, immune cells, and non-cellular components such as extracellular matrix (ECM), chemokines, cytokines, and other soluble circulating elements (4, 42). The ECM is an essential part of the TME, where collagen is one of the most abundant proteins. Several studies have demonstrated that collagen fibres play a significant role in the context of cancer (43). The development of tumour cells is closely associated with changes in the TME, reflected in the “hardening” of the tumour-associated stroma (4, 5). Collagen changes have been reported in invasive breast carcinoma, highlighting the significant role of this protein in tumour invasiveness (44, 45). A more detailed assessment of collagen characteristics enabled its use as a prognostic and predictive tool. In gastric and breast cancer, changes in the characteristics of collagen fibres correlate with survival (29, 46).

Here we demonstrate that collagen fibres are more organised in CMT when compared to BMT and NMT (Figure 3A). A previously published study by our group, evaluating samples of normal and neoplastic canine mammary tissue imaging by multiphoton microscopy, revealed that carcinomas present more organised collagen fibres compared to the normal mammary gland (29). Here, the BMT shows longer collagen fibres than the NMT (Figure 3B). CMT presents longer fibres as compared to BMT and CMTM. When comparing carcinoma groups, CMTM presents shorter collagen fibres as compared to CMT. Furthermore, the data indicate that CMT presents more linear fibres as compared to NMT, BMT, and CMTM (Figure 3C). We believe that the shorter fibre length in carcinomas may be associated with increased tumour aggressiveness and progression to metastatic disease.

Our previous work showed similar results (29). In this study, a detailed assessment of collagen fibre characteristics was performed in the benign, *in situ*, and invasive regions of CMT. We demonstrated that the invasive areas exhibited shorter collagen fibres compared to the *in situ* CMT areas. These findings indicate that during tumour progression, the length of collagen fibres increases as the neoplasm develops. However, during the invasion process, the fibre length cases decrease again when compared to malignant regions *in situ*. A possible explanation is the breakdown of these fibres by proteolytic enzymes such as metalloproteinases, which due to their pro-tumour activity, stimulate the invasion of neoplastic cells (29, 47, 48). Furthermore, we demonstrated that the shorter length of collagen fibres correlates with a greater number of these structures in the CMT and CMTM (Figure 4A). These data, combined with the increased fibre waviness in CMTM, provide further evidence to support the hypothesis that in invasive regions, collagen fibres are disrupted to facilitate lymphatic invasion and potentially establish metastatic disease.

Regarding the number of collagen fibres, the data show that BMT, CMT, and CMTM present a lower number of fibres as compared to NMT (Figure 3D). Among the neoplastic groups, we observed that the number of fibres is higher in BMT as compared to CMT and CMTM. Between the carcinomatous groups, no significant differences were observed for this parameter. These findings are explained by the morphological characteristics of mixed tumours during tumour progression. While in BMT there is an increased proliferation of the stromal component compared to the epithelial component, in carcinomatous groups epithelial proliferation is more prominent compared to stromal proliferation (1–3, 29, 32, 49). Furthermore, mixed tumours showed greater cell coverage when compared to BMT and consequently lower coverage of collagen fibres as compared to NMT. Among mixed tumours, we noticed that BMT has a lower cellular coverage and larger coverage of collagen fibres as compared to CMT and CMTM. These data are consistent with data from the literature. BMT can exhibit benign proliferation of epithelial components alone or both epithelial and mesenchymal (or stromal) components in equal proportions. Conversely, in CMTs, only the epithelial component is malignant, while the mesenchymal component may remain unchanged or exhibit benign proliferation. Considering these distinctions, we observe a predominance of epithelial proliferation in CMTs compared to BMTs, whereas stromal proliferation is more prominent in BMTs compared to CMTs (1–3, 29, 32).

Note that carcinomas present a larger number of collagen fibres compared to NMT and BMT, but a smaller area covered by fibres. This indicates that in NMT and BMT the fibres are spread out in the tissue in a disorganised way and there is a connection between these fibres. This occurs because collagen organises itself around cancer cells to initially contain neoplastic growth and, subsequently, guide these cells towards stromal invasion. During this organisational process, the fibres lose their interconnection. This information is important because PyFibre (see material and methods) quantifies the number of fibres based on individual fibre extraction and the area covered by fibres from the regions of the image occupied by fibres. Thus, if the extracted fibres are interconnected and disorganised in the tissue, the programme will quantify a smaller number of fibres and a larger area covered by fibres, as seen in NMT and BMT. On the other hand, when the extracted fibres are not interconnected and are organised in specific regions of the tissue, the programme will quantify a larger number of fibres and a smaller area covered by fibres, characteristics observed in carcinomas.

To evaluate the relationship between our findings and the CMT and CMTM immunohistochemistry data, we correlated the data obtained from multiphoton microscopy with the immunostaining data for Ki67, ER, PR, COX-2, and HER2. Immunohistochemistry is a crucial tool that allows the evaluation of essential molecular markers to determine prognostic and predictive factors in veterinary medicine (32, 33). The immunohistochemical panel recommended for the canine mammary gland includes the immunomarkers Ki67, ER, PR, and COX-2. Using these immunomarkers it is possible to define the immunophenotype of breast carcinoma and verify the expression of COX-2. Immunohistochemical analysis of our carcinoma samples revealed that the majority were classified as luminal A, indicating the presence of hormone receptors and a low rate of cell proliferation (Ki67 < 20%). These results are in line with literature findings, supporting the idea that canine mammary gland carcinomas of this subtype often have a favourable prognosis. The loss of differentiation and increased aggressiveness of neoplastic cells are associated with the decrease or loss of expression of hormone receptors, a higher proliferative rate, and increased expression of COX2. These characteristics are observed in more aggressive neoplasms (32, 50, 51). Therefore, an assessment of immunohistochemical markers should be performed to determine prognosis and treatment considerations (32).

Here we observed that strong expression of COX-2 in at least 30% of the tumour extent was more prominent in carcinomas with shorter collagen fibres (Figure 4B). COX-2 expression is associated with increased aggressiveness and worse prognosis, influencing cell proliferation, inhibition of apoptosis, induction of angiogenesis, and modulation of the immune system (52–55). Thus, COX-2 inhibition has been widely used in the treatment of several types of neoplasms in dogs, including mammary neoplasms (2, 3, 32, 52). Furthermore, the cell proliferation rate showed a moderate direct correlation with the number of collagen fibres in the CMT and CMTM (Figure 4C). These data indicate that a higher cell proliferation rate is associated with carcinomas with a larger number of collagen fibres. Ki67 is a protein found in proliferating cells, present in all phases of the cell cycle, except the G0 phase (56). In canine mammary neoplasms, Ki67 is a known prognostic marker. More aggressive neoplasms express marking larger than 20% and, when exceeded, correlate with a worse prognosis (32). In this context, we observed that carcinomas with a higher density of collagen fibres and shorter fibres present characteristics of more aggressive neoplasms, such as a high proliferation rate and increased expression of COX-2. These findings complement what was mentioned previously, as we observed that carcinomas that progressed to metastatic disease (CMTM) presented shorter and more wavy collagen fibres. These findings reinforce our hypothesis that in invasive areas, collagen fibres break and guide cells towards lymphatic vessels in hardened, organised collagen. This process favours metastasis to the lymph nodes.

We did not find statistically significant relationships between the characteristics of collagen fibres and the expression of hormone receptors and HER-2 expression. Possibly, the absence of other significant correlations may be related to the fact that our samples were limited to carcinomas in mixed tumours, known to have a favourable prognosis and high survival rates. However, we were able to demonstrate that modifications in collagen fibres can facilitate the occurrence of metastatic diseases even in carcinomas with a favourable prognosis.

## 5 Conclusion

Using second harmonic generation microscopy combined with two-photon excited fluorescence microscopy to evaluate collagen fibres revealed significant differences in collagen structure in canine mixed mammary tumours. The results made it possible to identify that carcinoma that evolved to metastatic disease (CMTM) presents shorter and more wavy collagen fibres when compared to CMT. In addition, carcinomas present a smaller area of fibre coverage, a larger area of cellular coverage, and a larger number of individual fibres as compared to NMT and BMT. Furthermore, we observed a correlation between strong COX-2 expression and a high rate of cell proliferation in carcinomas with a smaller area covered by cells and a larger number of individual fibres. These findings highlight the crucial role of collagen in tumour progression and the process of invasion and metastatic spread. This deeper understanding of collagen modifications provides valuable information about the involvement of this component in the behaviour of mammary carcinomas. Changes in collagen fibres are key determinants of macromolecular transport in tumours. Therefore, resistance to chemotherapy may be associated with significant differences in the collagen structure in canine mixed mammary tumours, since CMTM have shorter and more wavy collagen fibres when compared to carcinomas in mixed tumours with local disease. These specific characteristics were not evaluated in this study. However, we intend to include these analyses in our upcoming reviews.

## Data availability statement

The raw data supporting the conclusions of this article will be made available by the authors, without undue reservation.

## Ethics statement

The animal studies were approved by ‘Ethics Committee for Animal Use’ at the Federal University of Minas Gerais under number 251/2018. The studies were conducted in accordance with the local legislation and institutional requirements. Written informed consent was obtained from the owners for the participation of their animals in this study.

## Author contributions

AG: Data curation, Formal analysis, Investigation, Methodology, Writing – original draft. DT: Data curation, Methodology, Visualization, Writing – original draft. LR: Data curation, Investigation, Methodology, Writing – original draft. AP: Conceptualization, Methodology, Supervision, Writing – review & editing. GC: Conceptualization, Funding acquisition, Methodology, Supervision, Writing – review & editing.
